# Anti-malarial activity of a polyherbal product (Nefang) during early and established *Plasmodium* infection in rodent models

**DOI:** 10.1186/1475-2875-13-456

**Published:** 2014-11-25

**Authors:** Protus Arrey Tarkang, Faith A Okalebo, Lawrence S Ayong, Gabriel A Agbor, Anastasia N Guantai

**Affiliations:** Centre for Research on Medicinal Plants and Traditional Medicine, Institute of Medical Research and Medicinal Plants Studies (IMPM), P. O. Box 6163, Yaoundé, Cameroon; Department of Pharmacology and Pharmacognosy, University of Nairobi, P. O. Box 19676–00202, Nairobi, Kenya; Early Drug Discovery Programme, Institut Pasteur Korea, Sampyeong-dong 696, Bundang-gu, Seongnam-si, Gyeonggi-do South Korea; Public Health and Epidemiology Unit, Centre Pasteur du Cameroun, P. O. Box 1274, Yaoundé, Cameroon

**Keywords:** Medicinal Plants, Nefang, Acute toxicity, Malaria, In vivo antiplasmodial activity, Suppressive activity, Prophylactic activity, Curative activity, Combination phytotherapy

## Abstract

**Background:**

The emerging resistance of *Plasmodium* species to currently available anti-malarials remains a public health concern, hence the need for new effective, safe and affordable drugs. Natural products remain a reliable source of drugs. *Nefang* is a polyherbal anti-malarial of the Cameroonian folklore medicine with demonstrated *in vitro* antiplasmodial and antioxidant activities. It is composed of *Mangifera indica* (bark and leaf), *Psidium guajava, Carica papaya, Cymbopogon citratus, Citrus sinensis, Ocimum gratissimum* (leaves). This study aimed at investigating the suppressive, prophylactic and curative activities of Nefang in *Plasmodium* infected rodent models.

**Methods:**

Systemic acute oral toxicity of Nefang aqueous and ethanol extracts was assessed in mice up to a dose of 5,000 mgkg^−1^ body weight. BALB/c mice and Wistar rats were inoculated with *Plasmodium chabaudi chabaudi* and *Plasmodium berghei,* respectively, and treated with Nefang, the *Mangifera indica* bark/*Psidium guajava* combination and a *Psidium guajava* leaf aqueous extracts (75, 150, 300 and 600 mgkg^−1^ bwt). Their schizonticidal activity was then evaluated using the Peter’s 4-day suppressive test). The prophylactic and curative (Rane’s Test) activity of Nefang was also evaluated by determining the parasitaemia, survival time, body weight and temperature in pre-treated rodents.

**Results:**

Acute oral toxicity of the extract did not cause any observed adverse effects. Percent suppressions of parasitaemia at 600 mgkg^−1^ bwt were as follows (*P. berghei*/*P. chabaudi*): Nefang – 82.9/86.3, *Mangifera indica* bark/*Psidium guajava* leaf combination extract – 79.5/81.2 and *Psidium guajava* leaf – 58.9/67.4. Nefang exhibited a prophylactic activity of 79.5% and its chemotherapeutic effects ranged from 61.2 – 86.1% with maximum effect observed at the highest experimental dose.

**Conclusion:**

These results indicate that Nefang has excellent *in vivo* anti-malarial activities against *P. berghei* and *P. chabaudi*, upholding earlier *in vitro* antiplasmodial activities against multi-drug resistant *P. falciparum* parasites as well as its traditional use. Hence, Nefang represents a promising source of new anti-malarial agents.

**Electronic supplementary material:**

The online version of this article (doi:10.1186/1475-2875-13-456) contains supplementary material, which is available to authorized users.

## Background

Despite substantial efforts to control malaria in the last few decades, it remains one of the most prevalent infectious diseases globally. The emerging resistance of *Plasmodium* species to currently available drugs remains a public health concern [1]. Forty percent of the world’s population is exposed to malaria and there is a constant need for new anti-malarials. Historically, plants have had a remarkable role in therapeutics and were the principal source of drugs for many centuries. Quinine, isolated in 1820 from Cinchona species (Rubiaceae), is an illustrative example. Drugs in current use for malaria chemotherapy include artemisinin, from *Artemisia annua* (Asteraceae) of Chinese origin, and its semi-synthetic derivatives, artemether, artesunate and arteether [2]. A recently introduced plant-derived anti-malarial drug is atovaquone, a synthetic naphthoquinone based on lapachol. Lapachol, a prenylnaphtoquinone, was first isolated from *Tabebuia impetiginosa*, a South American member of the Bignoniaceae family [3]. Artemisinin-based combination therapy (ACT) is currently the most effective chemotherapy against *Plasmodium falciparum* malaria and the emergence of resistance would be a public health disaster in malaria endemic areas. Therefore, plants do not only provide valuable clues for finding new drugs, but may help to shift the drug discovery paradigm from finding new molecules to combining existing agents [4, 5].

The modern pharmaceutical industry was born from botanical medicine, but standardized synthetic combinatorial chemistry in drug discovery and high throughput screening (HTS) of potential drug targets have disconnected the historical link between plants and medicines. However, this has been rekindled by the small output of modern anti-malarial pharmaceutical research and development, which has stimulated new interest in the potential of natural compounds [6]. Hence natural products continue to provide new starting points in drug discovery.

There is a school of thought that biologically-derived secondary metabolites and synthetic compounds derived from them perform better as drugs than randomly synthesized compounds. Drug-derived parent molecules were present in primitive life forms and therefore co-evolved to interact with one another, thus granting direct ecological benefit to the producing organism, whether in competition for resources, avoiding predation or combating pathogens [7]. This co-evolution between different plants and/or metabolites within the same plant brings about synergy or potentiation which has been proven to achieve favourable results, such as enhanced efficacy, decreased dosage at equal or increased level of target inhibition, reduced or delayed development of drug resistance and simultaneous reduction of toxic effects [8]. Based on this paradigm, drug combination in anti-malarial chemotherapy has been adopted and is widely used as a strategy to monitor and prevent resistance [9].

*Nefang* is a polyherbal product composed of the aqueous extracts of *Mangifera indica* (bark and leaf), *Psidium guajava, Carica papaya, Cymbopogon citratus, Citrus sinensis* and *Ocimum gratissimum* (leaves). It is frequently used for the treatment of malaria in the South West Region of Cameroon. Ethnopharmacological studies confirmed its formulation and folk use [10]. Studies have been conducted to evaluate the *in vitro* and *in vivo* antioxidant properties of this polyherbal [11], which may play a role in curbing oxidative stress related with malaria infection. The *in vitro* antiplasmodial activity of this product and solvent extracts of its constituents have also been evaluated, showing good activities and synergistic potentials of some constituent extracts [12].

This study aimed at evaluating the in vivo suppressive, prophylactic and curative activities of *Nefang* in mice and rats models. Results obtained showed that Nefang exhibited excellent *in vivo* anti-malarial activities in rodents, consistent with its previously observed anti-*P. falciparum* activities.

## Methods

### Extraction of plant material

Fresh parts of the constituent plants of Nefang: bark and leaves of *Mangifera indica* (*MiB* and *MiL*, respectively), and leaves of *Psidium guajava* (*Pg*), *Carica papaya* (*Cp*), *Cymbopogon citratus* (*Cc*), *Citrus sinensis* (*Cs*), *Ocimum gratissimum* (*Og*) were harvested from their natural habitat in Cameroon between July and August 2011. Plant identification and voucher specimen referencing were done at the Institute of Medical Research and Medicinal Plants Studies (IMPM) herbarium in Yaoundé, Cameroon by a botanist. The freshly harvested plant parts were air dried and pulverized. Aqueous extraction was performed based on the traditional knowledge of preparation. Research evidence shows that ethanol extracts are as effective as the water extracts [13] and, therefore, ethanol extraction was also performed. Weighed quantities (1,000 g) of each plant part were exhaustively macerated in water (2.4 L) and ethanol (2.0 L) respectively for 4 h. Each of the macerate was transferred into a conical percolator for 72 h and the extracts were filtered [14]. Each ethanol filtrate was first concentrated using a rotary evaporator. The filtrates were then concentrated in an air oven at 60°C. The extracts were weighed and stored in labeled sealed plastic containers at 4°C until use to prevent decomposition.

### Experimental animals

Swiss albino mice (25 – 30 g) were used for the acute toxicity testing while BALB/c mice (20 – 25 g) and Wistar rats (160 – 180 g) were used for *in vivo* antiplasmodial activities testing. All experimental animals were housed under standard environmental conditions of temperature at 22-24°C under a 12 h dark–light cycle, and allowed free access to drinking water and standard pellet diet.

Ethical approval for the study was obtained from Kenyatta National Hospital/University of Nairobi Ethics and Research committee, Nairobi-Kenya (KNH-ERC/A/324 - 5/12/12), Institute of Medical Research and Medicinal Plants Studies Institutional Review Board (076/82-62/MINRESI/M000 – 01/06/12) and Institut Pasteur Korea-Institutional Animal Care and Use Committee (IACUC No. IPK 12009 – 29/10/12).

### Acute (single dose) oral toxicity testing

The acute oral toxicity of Nefang aqueous and ethanol extracts was evaluated according to the procedures outlined by the Organization for Economic Co-operation and Development [15]. Each crude extract was suspended in a vehicle (distilled water and corn oil for the aqueous and ethanol extracts respectively). Following a 4 h fasting period, mice were divided into groups of three. Extract doses were calculated in reference to mice body weight and each mouse was treated with a single oral dose of the extract.

The mice were dosed in a stepwise procedure using the fixed doses of 5, 50, 300, 1,200 and 2,000 mgkg^−1^ body weight (bwt) of the aqueous and ethanol extracts for each group of mice respectively. After each dose, the animals were observed for signs of toxicity for three hours. If there was no mortality or signs of toxicity at the highest dose, then the upper limit dose was used for the main test.

For the main test, a single high oral dose of 5,000 mgkg^−1^ bwt of each extract was administered to three male (Test 1) and three female (Test 2) mice in the treatment groups, whereas the control groups received the vehicle. Food was provided to the mice approximately an hour after treatment. The animals were observed 30 min after dosing, followed by hourly observation for 8 h and once a day for the next 13 days. Observations were systematically recorded for each animal. Surviving animals were weighed and visual observations for mortality, behavioural pattern, changes in physical appearance, injury, pain and signs of illness were conducted daily during the study period.

### Parasite infection of experimental animals

The chloroquine sensitive strain of *Plasmodium berghei* (strain ANKA) was generously donated by the Institute of Primate Research (IPR), Nairobi, Kenya, while the *Plasmodium chabaudi chabaudi* was obtained from the Centre for Neglected Diseases Drug Discovery (CND3), Institut Pasteur Korea, as cryo-frozen stock of parasitized red blood cells (PRBCs). The parasites were prepared through two cycles of passage of the PRBCs in rats and mice. Donors with parasitaemia level of 20-30% were sacrificed and blood collected by cardiac puncture into heparinized tubes. The blood was then diluted with phosphate buffered saline (PBS) based on parasitaemia level of each donor and the RBC count of normal mice and rats, such that 1 mL blood contained 5 × 10^7^ parasites. The experimental animals were each treated with 1 × 10^7^ PRBCs by intraperitoneal (ip) injection [16].

### Test for suppressive activity (Peter’s 4-day test)

The aqueous extracts that were selected for this study were Nefang, *Psidium guajava* (*Pg*) (i.e. the most active constituent aqueous extract) and *Mangifera indica* bark/*Psidium guajava* leaf (*MiB/Pg*) (i.e. the solvent extract combination that showed the most promising synergistic activity) [12]. The suppressive activities of these extracts were evaluated in early *P. chabaudi* and *P. berghei* infection in BALB/c mice and Wistar rats, respectively, using the method described by Knight and Peters [17]. Forty-five mice and forty-five rats were each randomly divided into fifteen groups of three each. On the first day (D0), the mice and rats were each infected with 10^7^*P. chabaudi* and *P. berghei,* respectively. Three hours later, the experimental groups of mice and rats were each treated orally with 10 mLkg^−1^ bwt of the drug or extract as follows: Group 1 (negative/vehicle control) - PBS, Group 2 (positive control) - chloroquine (10 mgkg^−1^), Group 3 (positive control) - pyrimethamine (30 mgkg^−1^), Groups 4 to 7 - Nefang*,* Groups 8 to 11 - *Pg* and Groups 12 to 15 - *MiB/Pg.* The aqueous plant extracts were each administered orally at a dose of 75, 150, 300 and 600 mgkg^−1^ respectively. Treatment was carried out for four consecutive days (D0 – D3). The body weight of each mouse were measured on the first day (D0) and on the fifth day (D4) using a sensitive digital analytical balance, while the body temperature was taken before infection and three hours after infection (D0) and then monitored daily to the fifth day (D4).

On the fifth day (D4), thin blood film was prepared from the tail blood of each experimental animal, fixed in methanol and stained with Giemsa to reveal parasitized erythrocytes. Parasitaemia was determined by light microscopy using a 100X objective lens and the following equation:


Average percentage chemosuppression was calculated as


where *A* is the average percentage parasitaemia in the negative control group and *B* is the average percentage parasitaemia in the test group.

### Test for prophylactic activity

The repository activity of Nefang was assessed using the method described by Peters [18]. The mice were randomly divided into seven groups of three BALB/c mice each. Group 1 (negative control) was treated with 10 mLkg^−1^ of PBS, group 2 and 3 (positive controls) - CQ (10 mgkg^−1^) and pyrimethamine (30 mg/kg^−1^), respectively, group 4 to 7 (extract test groups) - 75, 150, 300 and 600 mgkg^−1^ of Nefang, respectively. Administration of the extract and standard drugs continued for three consecutive days (D0 - D2). On the fourth day (D3), the mice were inoculated with 10^7^*P. berghei* infected red blood cells and the level of parasitaemia was assessed by blood smear 72 h later.

### Test for curative activity (Rane’s test)

The schizontocidal activity of Nefang on established infection was evaluated using the method described by Ryley and Peters [19]. Twenty-one rats were infected with *P. berghei* (10^7^) PRBCs by ip injection on the first day (D0). Seventy-two hours later (D3), the rats were randomly divided into seven groups of three rats each. Four groups of the rats were treated orally with 75, 150, 300 and 600 mgkg^−1^ bwt of Nefang respectively. The negative control group was treated with PBS while the two positive control groups were treated with CQ (10 mgkg^−1^) and artesunate (5 mgkg^−1^), respectively. Nefang and the standard drugs were each treated once daily for five days. Giemsa-stained thin smears were prepared from tail blood samples collected on each day of treatment to monitor parasitaemia level. The body weight and temperature were taken before infection (D0) and from the fourth day (D3) to the eighth day (D7) while the mean survival time (MST) of the rats in each treatment group was determined over a period of 29 days (D0 – D28) as follows;


### Statistical analysis

Data are expressed as mean ± standard deviation (SD) of the mean. Data were analysed using SPSS Version 20.0. Statistical significance testing was done using the one-way analysis of variance (ANOVA) followed by Neuman-Keuls multiple comparison test. P-values of less than 0.05 were considered statistically significant.

## Results

### Acute (single dose) oral toxicity testing

There were no observed adverse effects at all oral dose levels (5, 50, 300, 2000 mgkg^−1^ bwt) for all the aqueous and ethanol extracts of Nefang and its constituents. All the mice survived. Similarly, oral administration of the aqueous and ethanol extracts of Nefang and its constituents at 5,000 mgkg^−1^ bwt, had no toxic effects throughout the 14-day study period. None of the mice showed any signs of toxicity, such as changes on skin, eyes and mucus membranes, behavioural patterns, trembling, diarrhoea, falling of the fur, sleep or coma. No significant changes were observed in their body weights. The estimated maximum tolerable dose (MTD) was above 5,000 mgkg^−1^ bwt for all extracts tested.

### Evaluation of the suppressive activity (Peter’s 4-Day Test)

**Figure 1:**
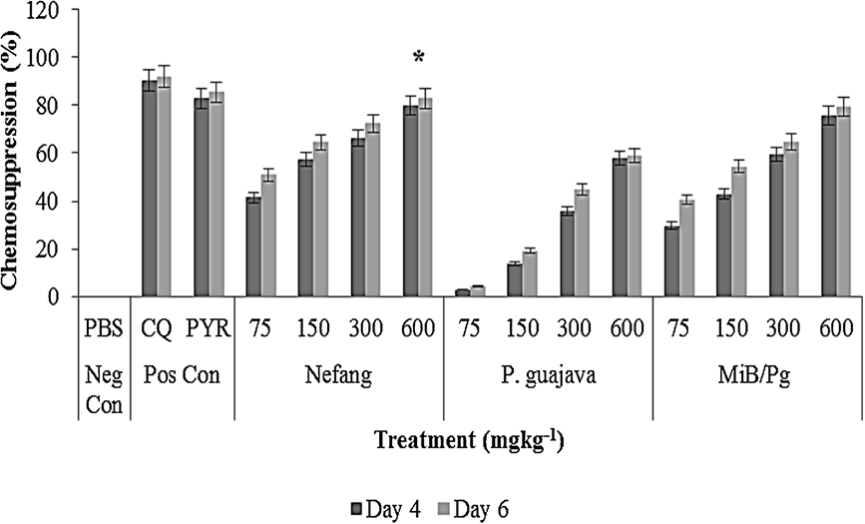
**Suppressive activity of Nefang and active solvent extracts on** ***P. berghei*** **infection in rats.** Each bar represents the Mean ± SD for each group of rats, n = 3. Neg Con – negative control; Pos Con – positive control; CQ – chloroquine; PYR – pyrimethamine. *Best chemosuppression compared to control.

**Figure 2:**
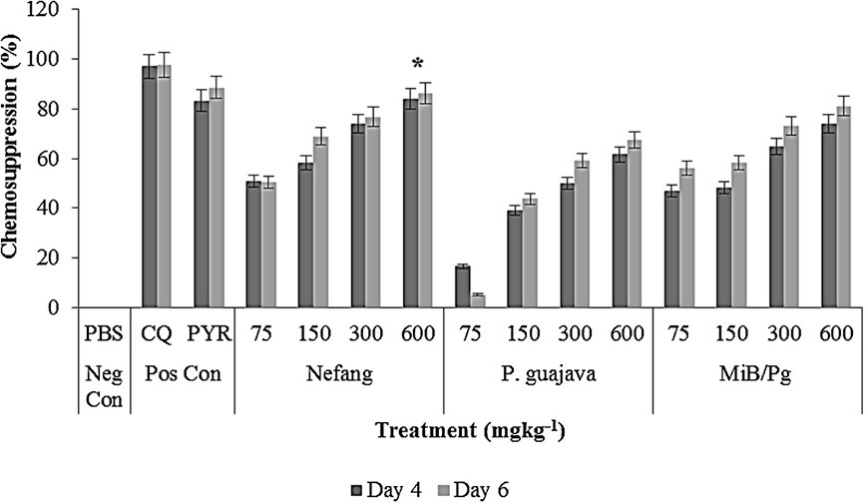
**Suppressive activity of Nefang and active solvent extracts on** ***P. chabaudi*** **infection in mice. Each bar represents the Mean ± SD for each group of rats, n =3.** Neg Con – negative control; Pos Con – positive control; *Best chemosuppression compared to control.

Nefang, *Psidium guajava* and *MiB/Pg* all showed dose-dependent chemosuppressive activities on parasitaemia. These effects were statistically significant (p < 0.001) relative to the vehicle treated group. At all doses, the suppressive activity in rats (Figure 1) and mice (Figure 2) were comparable. On day 6, at the highest experimental dose of 600 mgkg^−1^, percentage suppression of parasitaemia were as follows (*P. berghei*/*P. chabaudi*): Nefang - 82.9/86.3, *MiB/Pg* - 79.5/81.2, *Pg* - 58.9/67.4 as against CQ (10 mgkg^−1^) - 92.1/97.7 and pyrimethamine (30 mgkg^−1^) - 85.5/88.5. This shows that the *in vivo* activity of Nefang was comparable to that of pyrimethamine and better than for *MiB/Pg* and *Pg*. It was significantly (p < 0.05) lower than for CQ.

**Figure 3:**
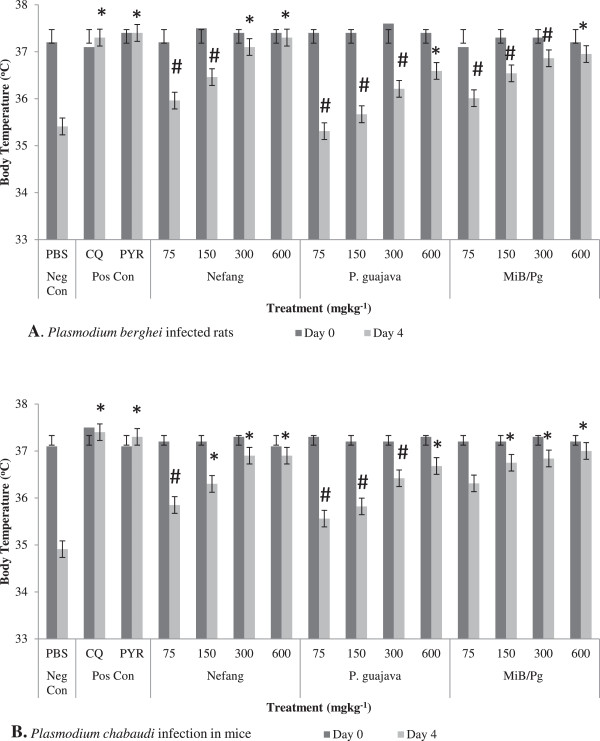
**Body temperature (Day 0 and Day 4) of Plasmodium infected animals treated with aqueous extract of Nefang and its active components in the 4-day suppressive test: (A)** ***Plasmodium berghei*** **infected rats (B)** ***Plasmodium chabaudi*** **infected mice.** *Compared to negative control (Neg Con); #Compared to positive control (Pos Con); Significant difference: * and #p < 0.05. CQ-chloroquine; PYR-pyrimethamine.

**Figure 4:**
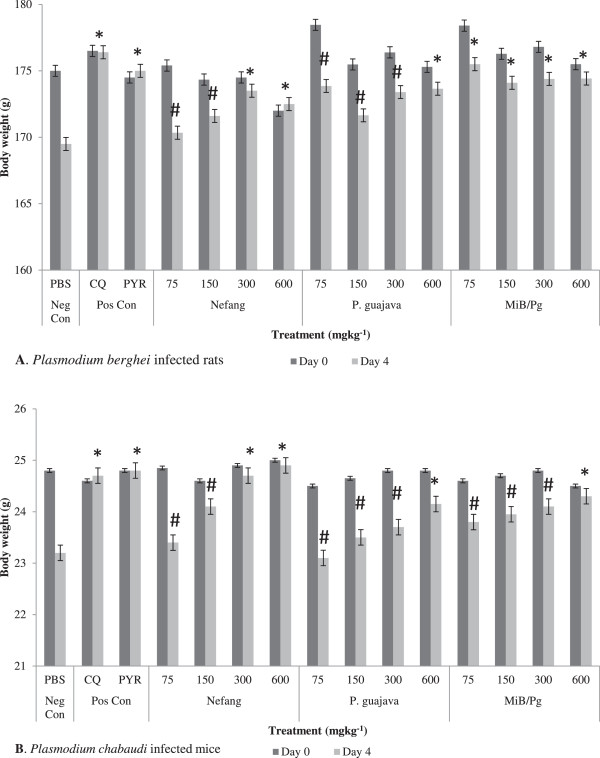
**Body weight (Day 0 and Day 4) of Plasmodium infected animals treated with aqueous extract of Nefang and its active components in the 4-day suppressive test: (A)** ***Plasmodium berghei*** **infected rats (B)** ***Plasmodium chabaudi*** **infected mice.** *Compared to negative control (Neg Con); #Compared to positive control (Pos Con); Significant difference: * and #p < 0.05. CQ-chloroquine; PYR-pyrimethamine.

Nefang (300 and 600 mgkg^−1^; p < 0.01) and *MiB/Pg* (600 mgkg^−1^; p < 0.05) caused significant attenuation of reduction in body temperature in both *P. berghei* infected rats and *P. chabaudi* infected mice in a dose-dependent manner (Figure 3). The effects of Nefang and *MiB/Pg* on the body temperature of experimental animals were comparable to those of the standard drugs CQ and PYR (Additional file 1). The extracts averted the loss of body weight associated with *Plasmodium* infection at same doses when compared to the control (Figure 4). No significant increases in weight were observed (see Additional file 2).

### Evaluation of the prophylactic activity

**Figure 5:**
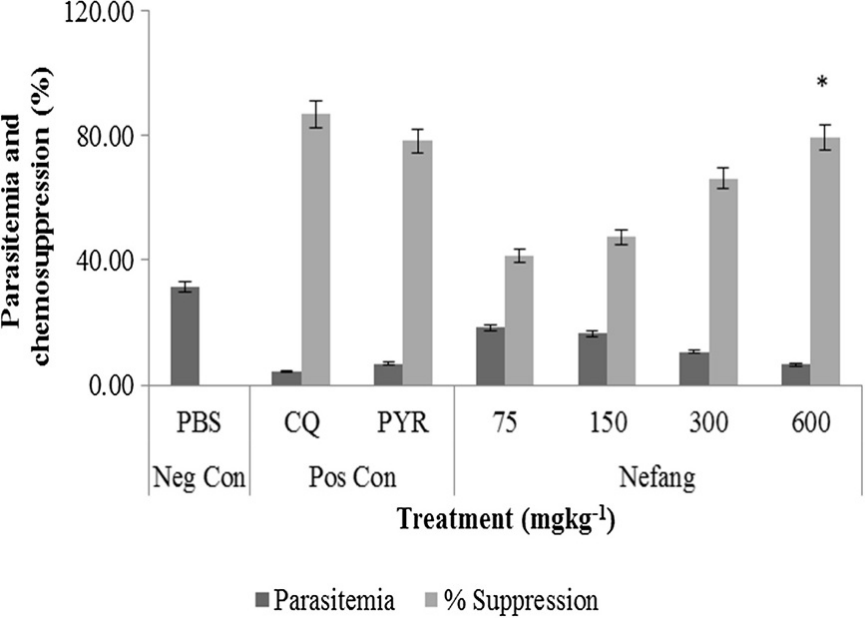
**Prophylactic activity of Nefang against early** ***P*** **.** ***berghei*** **infection in BALB/c mice.** *Compared to negative control (Neg Con); #Compared to positive control (Pos Con); Significant difference: p < 0.05. CQ-chloroquine; PYR-pyrimethamine.

Nefang aqueous extract showed a dose-dependent reduction of parasitaemia in experimental groups of mice. These reductions are statistically significant relative to the control. At the highest experimental dose of 600 mgkg^−1^, Nefang demonstrated a high antiplasmodial activity of 79.5%, which was very significant (p < 0.001) when compared to the vehicle. Though slightly lower than that exhibited by CQ (86.9%) at 10 mgkg^−1^, it was comparable to that exhibited by PYR (78.4%) at 30 mgkg^−1^ (Figure 5).

### Evaluation of the curative activity (Rane’s test)

**Figure 6:**
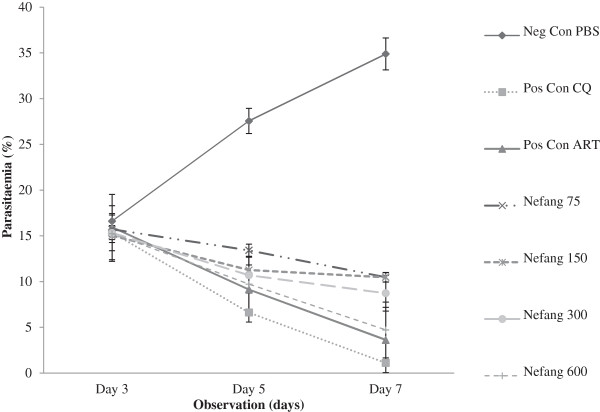
**Curative effect of Nefang aqueous extract against established** ***P. berghei*** **infection in Wistar rats. Each data point represents the Mean ± SD for each group of rats, n =3.** Neg Con-negative control; Pos Con-positive control; CQ-chloroquine; ART- artesunate.

**Figure 7:**
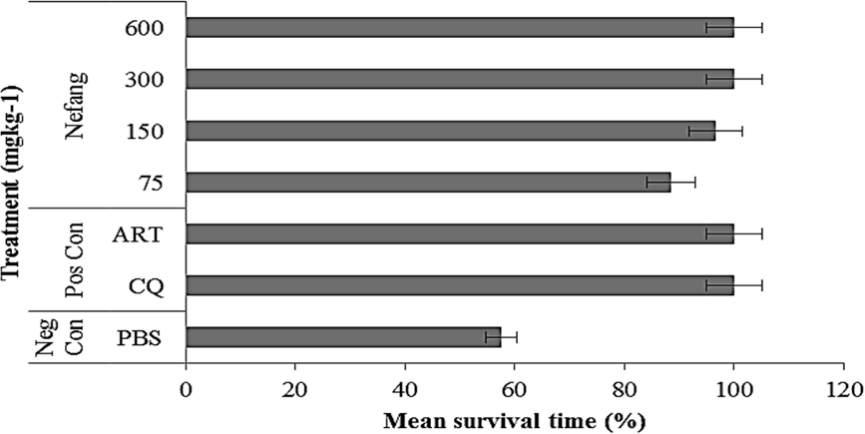
**Mean survival time of Wistar rats treated with Nefang aqueous extract during established** ***P*** **.** ***berghei*** **infection. Each bar represents the Mean ± SD for each group of rats, n =3.** Neg Con-negative control; Pos Con-positive control; CQ-chloroquine; ART-artesunate.

There was a dose-dependent reduction of parasitaemia in all Nefang experimental groups relative to the vehicle. These reductions were statistically significant (p < 0.001) when compared to the vehicle. The dose-dependent chemosuppression exhibited by Nefang at a dose of 600 mgkg^−1^ bwt was comparable to that of the standard drugs, CQ (10mgkg^−1^) and artesunate (5 mgkg^−1^) (Figure 6; see Additional file 3). Nefang also demonstrated a significant (p < 0.05) protective effect on the experimental rats as observed in the mean survival time of the animals, especially at the highest dose with all animals surviving through the experiment (Figure 7; see Additional file 4).

**Figure 8:**
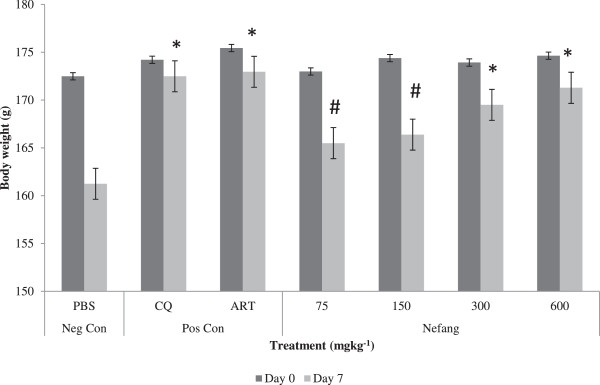
**Effect of Nefang aqueous extract on the body weight of** ***P. berghei*** **infected rats during established infection (Rane’s Test).** *Compared to negative control (Neg Con); #Compared to positive control (Pos Con); Significant difference: p < 0.05). CQ-chloroquine; ART-artesunate.

**Figure 9:**
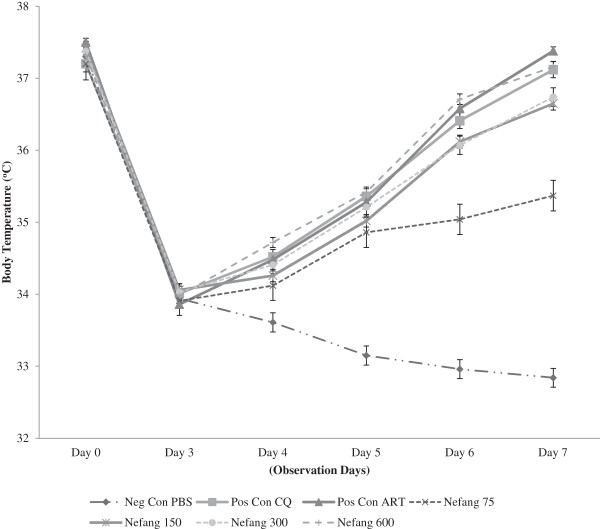
**Effect of Nefang aqueous extract on the body temperature of** ***P. berghei*** **infected rats during established infection (Rane’s Test).** Each data point represents the Mean ± SD for each group of rats, n =3. Neg Con-negative control; Pos Con-positive control; CQ-chloroquine; ART-artesunate.

Reduction in body weight of infected animals was significantly (p < 0.05) prevented by Nefang at doses of 300 and 600 mgkg^−1^. This activity was comparable to that of the standard drugs, CQ and artesunate (Figure 8). At same doses, Nefang significantly (p < 0.001) prevented the reduction in body temperature of infected animals and this was comparable to that of the standard drugs (Figure 9).

## Discussion

The *in vivo* antiplasmodial activities of the aqueous extract of Nefang and its active components, *Pg* and *MiB/Pg,* were investigated by evaluating the chemosuppression during early infection, while Nefang alone was evaluated during established infection using standard animal models. *In vivo* models are usually employed in anti-malarial studies because they take into account the possible prodrug effect and probable involvement of the immune system in eradication of the pathogen [20]. During early infection, Peter’s 4-Day suppressive test was used to evaluate schizontocidal activity while the repository test was used to study the prophylactic activity. Rane’s test was used to study curative ability during established infection. In all methods, determination of percent inhibition of parasitaemia was the most reliable parameter. A mean parasitaemia level that is ≤90% of that of the vehicle treated animals usually indicates that the test compound is active [21]. In the 4-day suppressive activity, Nefang and *MiB/Pg* significantly reduced parasitaemia (in both *Plasmodium spp*) in animal models in a dose-dependent manner, with Nefang exhibiting anti-malarial activities comparable to that of the standard drugs tested. The repository test revealed the same dose-dependent chemosuppression by Nefang. In the curative activity, the dose-dependent activity of Nefang at the highest experimental dose was observed from Day 2 of treatment. Though its activity was lower than that of CQ, it was comparable to that of ART. Furthermore, we observed that the survival time of Nefang-treated animals was prolonged in a dose-dependent manner (Figure 7). Nefang caused a dose-dependent reduction of pyrexia and loss of body weight in infected animals. Body weight loss and temperature reduction are hallmarks of malaria infection in animal models [22], suggesting that an effective plant-derived anti-malarial agent should prevent body weight loss in *Plasmodium* infected animals. This dose-dependent preventive activity indicates that Nefang does not have any adverse effect in experimental animals at the doses tested and as observed in our oral acute toxicity studies.

In acute toxicity testing, doses higher than 5,000 mgkg^−1^ bwt are generally not considered as dose related, which is in accordance with the Organization for Economic Corporation and Development (OECD) Guidance Document for Acute Oral Toxicity Testing [23, 15]. Compounds with LD_50_ values lower than 2,000 mgkg^−1^ bwt are generally considered to be relatively safe, because values above this are non-classified. This signifies that Nefang and its constituent aqueous and ethanol extracts can be considered as non-toxic at acute oral administration since the extracts were well tolerated and no observed adverse effect levels were >5,000 mgkg^−1^. These results are consistent with earlier reports on the cytotoxicity of Nefan*g* [12].

Unlike in humans, increase in parasitaemia levels in rodent models usually results in decreased metabolic rates and a consequent decrease in body temperatures [24], which might result in death. An ideal anti-malarial agent would, therefore, prevent this occurrence, an effect observed in Nefang-treated animals. Taken together, these results confirm that Nefang has therapeutic activity against established infection and further confirm the in vitro antiplasmodial activities reported earlier. At the highest dose (600 mgkg^−1^) tested, Nefang exhibited an *in vivo* suppression of parasitaemia of >80%, prophylactic activity of 79.5% and chemotherapeutic effects of 60 – 80%. *In vivo* antiplasmodial activity can be classified as moderate, good, and very good if an extract displayed percentage parasitaemia suppression equal to or greater than 50% at a dose of 500, 250 and 100 mgkg^−1^ per day, respectively [25], suggesting, therefore, that Nefang has very good activity. This also suggests that the overall anti-malarial activity of the synergistic and additive components identified during the interaction studies [12], over-shadowed the antagonistic interactions. Synergy between different constituents of extracts has been documented for anti-malarial and other pharmacological activities [26, 27].

Earlier phytochemical screening of the constituent plant extracts of Nefang showed the presence of alkaloids, anthocyanins, flavonoids, phenols, saponins, tannins, triterpenes and sterols [12], suggesting that the anti-malarial activity of Nefang cannot be attributed only to the active antiplasmodial compounds in the constituent plant extracts. In our earlier *in vitro* antiplasmodial studies on the constituent plant extracts of Nefang, only *M. indica* (bark and leaf) and *P. guajava* showed good activities while the rest showed very weak activities or inactivity. However, there have been reports of good *in vivo* antiplasmodial and anti-malarial activities exhibited by *C. papaya* [28], *C. citratus* [29] and *O. gratissimum* [30], which are constituent plants of Nefang. As earlier reported, many anti-malarial herbal remedies may exert their anti-infective effects not only by directly affecting the pathogen, but also by indirectly stimulating natural and adaptive defense mechanisms of the host by other mechanisms. Therefore, extracts that can stimulate innate and/or adaptive immunity may be able to contribute to prophylaxis and treatment not only for malaria but for other diseases as well [31, 32]. This suggests that a combination of the biological activities of the constituent plant extracts of Nefang results in an enhanced overall anti-malarial activity of Nefang. Therefore, an understanding of the underlying pharmacodynamic or pharmacokinetic mechanisms of this action would be very important towards understanding the anti-malarial potentials of Nefang.

## Conclusion

This study provides evidence that Nefang is safe and possesses good *in vivo* suppressive, prophylactic and curative activities against *Plasmodium* species. These findings uphold earlier in vitro antiplasmodial activities and confirm the synergistic interactions between its constituents plant extracts. It further suggests that the observed *in vivo* anti-malarial activity of Nefang may result from the synergistic biological activities of its constituent plant extracts. Hence, Nefang represents a promising source of anti-malarial agents for downstream clinical development.

## Electronic supplementary material

Additional file 1: **Body temperature (Day 0 and Day 4) of Plasmodium infected animals treated with aqueous extract of Nefang and its active components in the 4-day suppressive test.** (DOCX 16 KB)

Additional file 2: **Body weight (Day 0 and Day 4) of Plasmodium infected animals treated with aqueous extract of Nefang and its active components in the 4-day suppressive test.** (DOCX 16 KB)

Additional file 3: **Effect of Nefang aqueous extract on the body weight of** ***P. berghei*** **infected rats during established infection (Rane’s Test).** (DOCX 15 KB)

Additional file 4: **Effect of Nefang aqueous extract on the body temperature of** ***P. berghei*** **infected rats during established infection (Rane’s Test).** (DOCX 15 KB)
