# Supplementary material for: Anti-malarial activity of a polyherbal product (Nefang) during early and established Plasmodium infection in rodent models
Source: Malar J. 2014 Nov 25;13:456. doi: 10.1186/1475-2875-13-456 (PMC4251988; doi:10.1186/1475-2875-13-456)
Supplement: Supplementary file 1 — Additional file 1: Body temperature (Day 0 and Day 4) of Plasmodium infected animals treated with aqueous extract of Nefang and its active components in the 4-day suppressive test. (DOCX 16 KB) [file 12936_2014_3607_MOESM1_ESM.docx]

**Additional file 1. Body temperature (Day 0 and Day 4) of *Plasmodium* infected animals treated with aqueous extract of *Nefang* and its active components in the 4-day suppressive test**

| **Extract** | **Dose**  **(mgkg^-1^)** | **Body Temp (^o^C)** (x̄ ± SD, n=3) | | | |
| --- | --- | --- | --- | --- | --- |
|  |  | ***P. berghei* infection in rats** | | ***P. c. chabaudi* infection in mice** | |
|  |  | **D0** | **D4** | **D0** | **D4** |
| **Negative Control** | 0 | 37.20 ± 0.16 | 35.41 ± 0.38 | 37.10 ± 0.41 | 34.91 ± 0.17 |
| **Positive Control (CQ)** | 10 | 37.10  ± 0.13 | 37.30  ± 0.21***^2^** | 37.50  ± 0.31 | 37.40  ± 0.09***^2^** |
| **Positive Control (PYR)** | 30 | 37.40  ± 0.19 | 37.40  ± 0.26***^2^** | 37.10  ± 0.11 | 37.30  ± 0.25***^2^** |
| ***Nefang*** | 75 | 37.20  ± 0.18 | 35. 96  ± 0.35**^#2^** | 37.20  ± 0.24 | 35.85  ± 0.31**^#2^** |
|  | 150 | 37.50  ± 0.47 | 36.46  ± 0.31**^#1^** | 37.20  ± 0.44 | 36.30  ± 0.53***^1^** |
|  | 300 | 37.40  ± 0.31 | 37.10  ± 0.16***^2^** | 37.30  ± 0.41 | 36.90  ± 0.18***^2^** |
|  | 600 | 37.40  ± 0.44 | 37.30  ± 0.39***^2^** | 37.10  ± 0.30 | 36.90  ± 0.29***^2^** |
| ***Pg*** | 75 | 37.40  ± 0.36 | 35.31  ± 0.48**^#2^** | 37.30  ± 0.32 | 35.56  ± 0.44**^#2^** |
|  | 150 | 37.40  ± 0.51 | 35.67  ± 0.52**^#2^** | 37.20  ± 0.52 | 35.82  ± 0.38**^#2^** |
|  | 300 | 37.60  ± 0.29 | 36.21  ± 0.52**^#1^** | 37.20  ± 0.33 | 36.42  ± 0.46**^#2^** |
|  | 600 | 37.40  ± 0.47 | 36.59  ± 0.15***^1^** | 37.30  ± 0.22 | 36.68  ± 0.26***^1^** |
| ***MiB/Pg*** | 75 | 37.10  ± 0.21 | 36.01  ± 0.33**^#1^** | 37.20  ± 0.21 | 36.31  ± 0.33 |
|  | 150 | 37.30  ± 0.24 | 36.54  ± 0.29**^#1^** | 37.20  ± 0.41 | 36.75  ± 0.16***^1^** |
|  | 300 | 37.30  ± 0.39 | 36.86  ± 0.37***^1^** | 37.30  ± 0.38 | 36.84  ± 0.19***^1^** |
|  | 600 | 37.20  ± 0.22 | 36.95  ± 0.26***^2^** | 37.20  ± 0.18 | 37.00  ± 0.23***^2^** |

******* *= compared to negative (-ve) control,* ***^#^*** *= to positive (+ve) control;*

*Significant difference:* ***^1^****= p<0.05,* ***^2^****=p<0.001.*
