# Supplementary material for: Anti-malarial activity of a polyherbal product (Nefang) during early and established Plasmodium infection in rodent models
Source: Malar J. 2014 Nov 25;13:456. doi: 10.1186/1475-2875-13-456 (PMC4251988; doi:10.1186/1475-2875-13-456)
Supplement: Supplementary file 2 — Additional file 2: Body weight (Day 0 and Day 4) of Plasmodium infected animals treated with aqueous extract of Nefang and its active components in the 4-day suppressive test. (DOCX 16 KB) [file 12936_2014_3607_MOESM2_ESM.docx]

**Additional file 2. Body weight (Day 0 and Day 4) of *Plasmodium* infected animals treated with aqueous extract of *Nefang* and its active components in the 4-day suppressive test**

| **Extract** | **Dose**  **(mgkg^-1^)** | **Body Weight (g)** (x̄ ± SD, n=3) | | | |
| --- | --- | --- | --- | --- | --- |
|  |  | ***P. berghei* infection in rats** | | ***P. c. chabaudi* infection in mice** | |
|  |  | **D0** | **D4** | **D0** | **D4** |
| **Negative Control** | 0 | 175.00 ± 2.81 | 169.50 ± 2.26 | 24.80 ± 0.12 | 23.20 ± 0.18 |
| **Positive Control (CQ)** | 10 | 176.50  ± 3.32 | 176.40  ± 3.48***^2^** | 24.60  ± 0.16 | 24.70  ± 0.21***^2^** |
| **Positive Control (PYR)** | 30 | 174.50  ± 2.43 | 175.00  ± 1.89***^2^** | 24.80  ± 0.19 | 24.80  ± 0.27***^2^** |
| ***Nefang*** | 75 | 175.40  ± 4.81 | 170.34  ± 6.21**^#2^** | 24.85  ± 0.11 | 23.40  ± 0.18**^#1^** |
|  | 150 | 174.34  ± 5.39 | 171.60  ± 4.32**^#1^** | 24.60  ± 0.29 | 24.10  ± 0.32***^1^** |
|  | 300 | 174.50  ± 3.96 | 173.50  ± 2.73***^1^** | 24.90  ± 0.23 | 24.70  ± 0.16***^2^** |
|  | 600 | 172.00  ± 2.85 | 172.50  ± 3.17***^2^** | 25.00  ± 0.18 | 24.90  ± 0.23***^2^** |
| ***Pg*** | 75 | 178.46  ± 9.37 | 173.86  ± 7.45**^#2^** | 24.50  ± 0.33 | 23.10  ± 0.36**^#2^** |
|  | 150 | 175.48  ± 6.59 | 171.65  ± 5.59**^#2^** | 24.65  ± 0.27 | 23.50  ± 0.41**^#1^** |
|  | 300 | 176.39  ± 9.38 | 173.40  ± 6.71**^#1^** | 24.80  ± 0.38 | 23.70  ± 0.25**^#1^** |
|  | 600 | 175.30  ± 7.21 | 173.65  ± 5.49***^1^** | 24.80  ± 0.22 | 24.15  ± 0.34***^1^** |
| ***MiB/Pg*** | 75 | 178.40  ± 6.73 | 175.50  ± 4.28**^#1^** | 24.60  ± 0.22 | 23.80  ± 0.47 |
|  | 150 | 176.28  ± 7.69 | 174.10  ± 7.12**^#1^** | 24.70  ± 0.35 | 23.95  ± 0.51 |
|  | 300 | 176.80  ± 7.16 | 174.39  ± 5.49***^1^** | 24.80  ± 0.44 | 24.10  ± 0.28***^1^** |
|  | 600 | 175.50  ± 4.65 | 174.42  ± 8.19***^2^** | 24.50  ± 0.17 | 24.30  ± 0.35***^2^** |

******* *= compared to negative (-ve) control,* ***^#^*** *= to positive (+ve) control;*

*Significant difference:* ***^1^****= p<0.05,* ***^2^****=p<0.001.*
