# Supplementary material for: Anti-malarial activity of a polyherbal product (Nefang) during early and established Plasmodium infection in rodent models
Source: Malar J. 2014 Nov 25;13:456. doi: 10.1186/1475-2875-13-456 (PMC4251988; doi:10.1186/1475-2875-13-456)
Supplement: Supplementary file 3 — Additional file 3: Effect of Nefang aqueous extract on the body weight of P. berghei infected rats during established infection (Rane’s Test). (DOCX 15 KB) [file 12936_2014_3607_MOESM3_ESM.docx]

**Additional file 3. Effect of *Nefang* aqueous extract on the body weight of *P.* *berghei* infected rats during established infection (Rane’s Test)**

| **Treatment** | **Dose**  **(mgkg^-1^)** | **Experimental Period (Days)** | |
| --- | --- | --- | --- |
|  |  | **Body weight (g)** (x̄ ± SD, n=3) | |
|  |  | **D0** | **D7** |
| **Negative Control** | - | 172.48 ± 4.69 | 161.24 ± 4.31 |
| **Positive Control (CQ)** | 10 | 174.21 ± 2.98 | 172.48 ±1.29***^1^** |
| **Positive Control (ART)** | 5 | 175.44 ± 3.24 | 172.95 ±3.21***^1^** |
| ***Nefang*** | 75 | 172.98 ± 4.37 | 165.49 ± 5.72**^#1^** |
|  | 150 | 174.38 ± 6.38 | 166.38 ± 4.38**^#1^** |
|  | 300 | 173.92 ± 3.87 | 169.49 ± 3.25***^1^** |
|  | 600 | 174.64 ± 5.40 | 171.28 ± 2.93***^1^** |

** = compared to negative control,* ***^#^*** *= to positive control*

*Significant Difference -* ***^1^*** *= p<0.05;* ***^2^*** *=p<0.001*

*CQ = chloroquine, ART = artesunate*
