# Supplementary material for: Anti-malarial activity of a polyherbal product (Nefang) during early and established Plasmodium infection in rodent models
Source: Malar J. 2014 Nov 25;13:456. doi: 10.1186/1475-2875-13-456 (PMC4251988; doi:10.1186/1475-2875-13-456)
Supplement: Supplementary file 4 — Additional file 4: Effect of Nefang aqueous extract on the body temperature of P. berghei infected rats during established infection (Rane’s Test). (DOCX 15 KB) [file 12936_2014_3607_MOESM4_ESM.docx]

**Additional file 4. Effect of *Nefang* aqueous extract on the body temperature of *P.* *berghei* infected rats during established infection (Rane’s Test)**

| **Treatment** | **Dose**  **(mgkg^-1^)** | **Experimental Period (Days)** | | | | | |
| --- | --- | --- | --- | --- | --- | --- | --- |
|  |  | **Body Temperature (^o^C)** (x̄ ± SD, n=3) | | | | | |
|  |  | **D0** | **D3** | **D4** | **D5** | **D6** | **D7** |
| **Negative Control** | - | 37.30  ± 0.31 | 33.94  ± 0.98 | 33.61  ± 1.08 | 33.15  ± 0.74 | 32.96  ± 0.53 | 32.84  ± 0.29 |
| **Positive Control (CQ)** | 10 | 37.20  ± 0.86 | 34.01  ± 0.83 | 34.52  ± 0.28 | 35.36  ± 0.38 | 36.41  ± 0.33 | 37.12  ± 0.24**^2^** |
| **Positive Control (ART)** | 5 | 37.50  ± 0.92 | 33.86  ± 0.17 | 34.48  ± 0.34 | 35.28  ± 0.27 | 36.58  ± 0.41 | 37.38  ± 0.21***^2^** |
| ***Nefang*** | 75 | 37.20  ± 1.12 | 33.91  ± 0.71 | 34.12  ± 0.32 | 34.86  ± 0.24 | 35.04  ± 0.28 | 35.37  ± 0.18**^#1^** |
|  | 150 | 37.42  ± 0.49 | 34.06  ± 0.53 | 34.26  ± 0.22 | 35.02  ± 0.17 | 36.12  ± 0.30 | 36.65  ± 0.26**^#1^** |
|  | 300 | 37.38  ± 0.94 | 34.03  ± 0.55 | 34.41  ± 0.74 | 35.21  ± 0.54 | 36.07  ± 0.15 | 36.74  ± 0.11***^2^** |
|  | 600 | 37.32  ± 1.26 | 33.98  ± 0.26 | 34.72  ± 0.23 | 35.42  ± 0.18 | 36.71  ± 0.62 | 37.16  ± 0.35***^2^** |

** = compared to negative control,* ***^#^*** *= to positive control*

*Significant Difference -* ***^1^*** *= p<0.05;* ***^2^*** *=p<0.001*

*CQ = chloroquine, ART = artesunate*
